# Vernix Caseosa Peritonitis as a Rare Cause of Acute Abdomen After Cesarean Section

**DOI:** 10.7759/cureus.17953

**Published:** 2021-09-14

**Authors:** Nuaman A Danawar, Ihab A ALmosalami, Olfa El Amine Elhadj, Raheel Anis, Ahmad Bubshait

**Affiliations:** 1 Department of General Surgery, Security Forces Hospital (SFHD), Dammam, SAU; 2 Department of Surgery, Security Forces Hospital (SFHD), Dammam, SAU; 3 Department of Histopathology, Security Forces Hospital (SFHD), Dammam, SAU

**Keywords:** amniotic fluid, vernix caseosa, peritonitis, vernix caseosa peritonitis, caesarean section, pregnancy, acute abdomen, laparotomy, laparoscopy, case report

## Abstract

Although Vernix caseosa peritonitis (VCP) is a rare complication of cesarean section (CS). It possesses a high rate of morbidity and its exact underlying pathology is not fully understood yet. However, it is assumed that the leakage of amniotic fluid into the maternal abdominal cavity triggers the inflammatory process. We discussed herein a 25-year-old patient who developed acute peritonitis three days after cesarean section. CT abdomen showed multiple fluid collections with no obvious other pathologies. At laparotomy, we found cheesy exudates covering the peritoneal surface of the abdominal viscera and multiple turbid fluid collections, but no bowel, uterine, or any another organ injury could be identified. Abdominal washing was done and, as the appendix was queried, an appendectomy was added. Histopathology study of the omental biopsy revealed mixed inflammatory infiltrate, fibrin, fetal hair, and squames. These findings suggest the diagnosis of VCP, but the removed appendix was normal. The postoperative course was long and complicated. A few cases of VCP were reported in the world; the majority of these cases are from the USA and a few are from the UK. According to the information we have, our case is the first reported case of VCP in Saudi Arabia. Typically, VCP is manifested as acute abdomen hours to weeks after CS or vaginal delivery. As VCP is usually mistaken for the other causes of acute peritonitis, and the diagnosis is only established by the histopathology examination of peritoneal or omental biopsies, the case is commonly managed by urgent laparotomy or laparoscopy with the removal of suspected organs, which are later confirmed to be normal on histopathology study. The typical intraoperative findings are adhesive exudate, white or cheese-like membranes covering the intraabdominal viscera, and fluid collections, but no visceral injuries can be identified. Therefore, it is crucial to include the VCP in the differential diagnosis of the acute abdomen after CS or vaginal delivery to avoid unnecessary laparotomy and removal of normal organs.

## Introduction

Vernix caseosa (VC) is a material that covers the surface body of a human neonate. The VC is specific to human babies; it consists of a cheesy sebaceous substance, soft hair, and epithelial squames of the baby skin [1]. VC has numerous functions that enable the newborn to overcome the new environment after delivery.

VCP is a rare but critical complication after CS or vaginal delivery. VCP is hypothesized to be due to the dribble of the fetal amniotic fluid or meconium into the maternal abdominal cavity [2]. VCP is of interest because it presents as an acute abdomen within hours to weeks after a straightforward CS or vaginal delivery [3-4]. The greatest challenge is that the VCP is confused with the common causes of acute abdomen and is a leading cause of unnecessary exploratory laparotomy or laparoscopy and surgical removal of seemingly involved abdominal organs such as the appendix, ovary, Fallopian tubes, and colon; finally, these organs are proved to be normal on histopathology examination [5-6]. VCP is not included in surgical or obstetric textbooks, and what we know about VCP is mainly based on case reports. Hence, many physicians and pathologists are not aware of this case, which leads to unnecessary laparotomy.

To our best of knowledge, this is the first reported and published case of VCP in Saudi Arabia. This study set out to increase awareness among obstetrics, pathologists, and surgeons about this rare complication after CS and to raise the point that not all cases of acute peritonitis after CS need laparotomy.

## Case presentation

**Figure 1:**
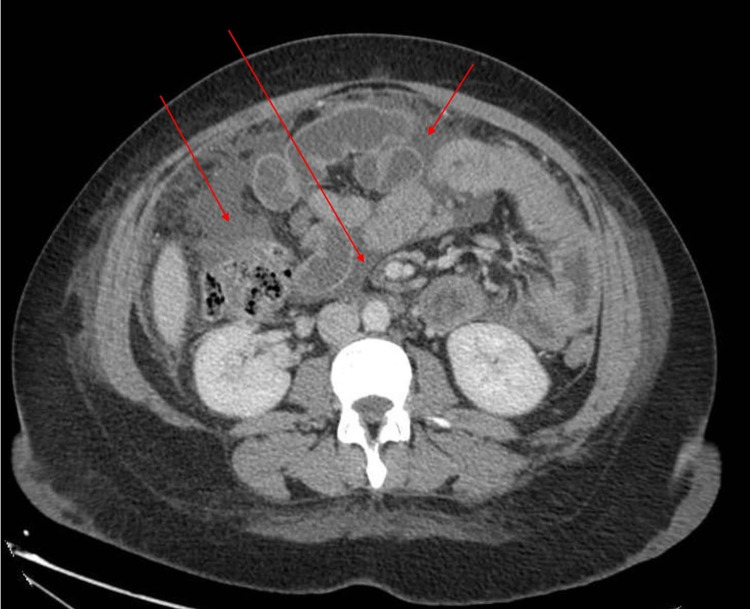
Axial CT abdomen demonstrating multiple intra-abdominal collections among bowel loops (red arrows)

A 25-year-old Saudi woman, gravida 1, para 0, has no past medical or surgical history of note. She has been on regular prenatal follow up since the 27 th week of gestation. In the 37th gestational week, the patient was admitted through the obstetric clinic for labor induction due to fetal growth restriction. A straightforward emergency CS was done because of fetal distress and failure of labor progression on medical induction. The first two post-CS days were unremarkable, and she was planned discharge possibly on the next day. On postop day three, the patient developed abdominal and chest pain with tachycardia 110/minute and shortness of breath. The clinical suspicion of pulmonary embolism was excluded by chest CT that only showed bilateral pleural and pericardial effusion (Figure 1). The on-call general surgery team (GST) was consulted.

On examination, her vital signs were as follows: blood pressure 122/88 mmHg, body temperature 38.4°C, and pulse rate 130 beats per minute, 30 breaths per minute. Her symptoms were heart palpitations, chest pain, and dyspnea. Physical examination of the abdomen revealed generalized abdominal guarding and rebound tenderness.

**Figure 2:**
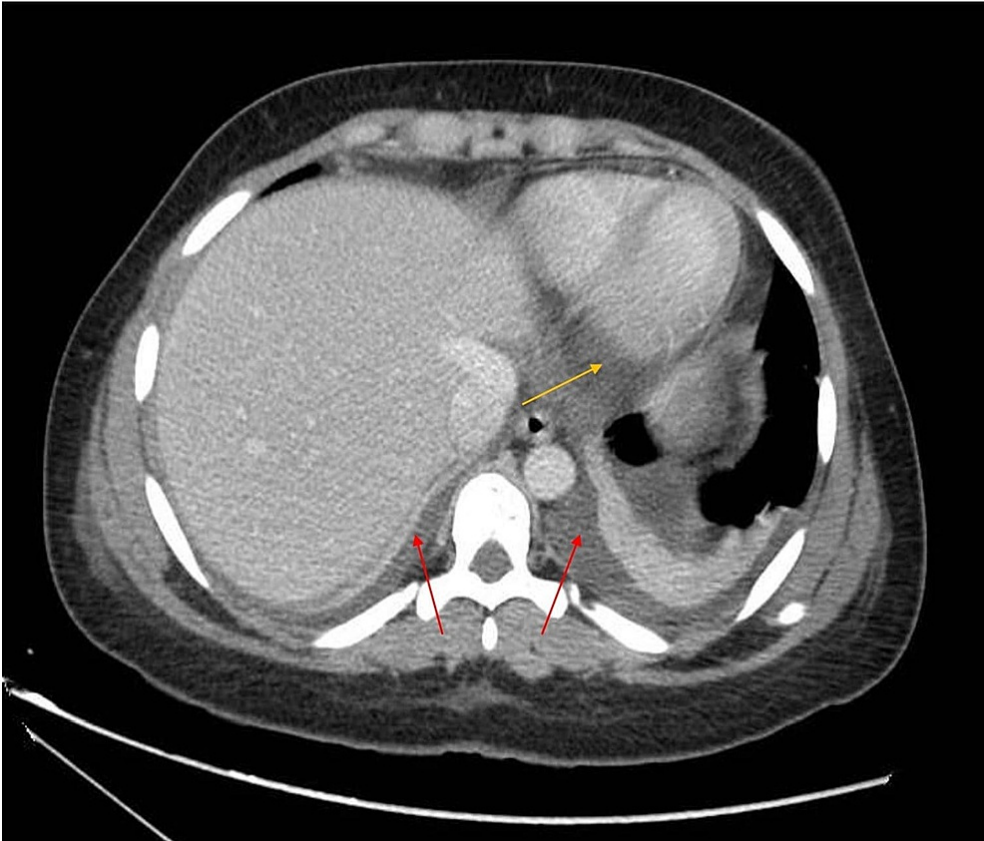
CT chest showing pericardial (yellow arrow) and bilateral pleural effusion (red arrows)

**Figure 3:**
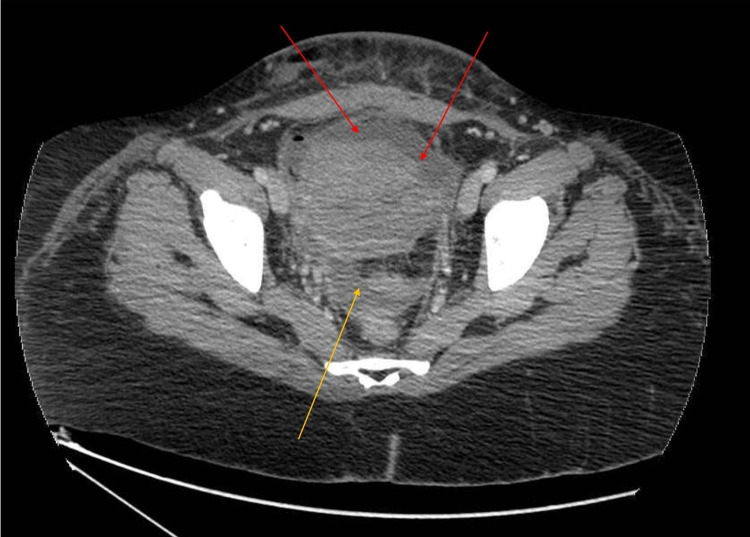
CT abdomen revealing pre-uterine (red arrows) and pelvic collections (yellow arrows)

Intravenous contrast-enhanced CT abdomen showed multiple interloop, subhepatic, pelvic, and pre-uterine collections without obvious contrast leak or organs injuries (Figures 2-3). The clinical impression was sepsis status post-CS surgery, hence conservative treatment was recommended by a multidisciplinary team (Ob-Gyn, pulmonology, and GST). On day six, despite the maximum medical and supportive treatment (ceftriaxone 2 g IV, chest physiotherapy, nebulizers), the patient failed to improve and developed a significant diffuse abdominal tenderness, rebound tenderness, high fever, and leukocytosis. Because of the failure of conservative treatment, the clinical suspension of visceral injury was raised, and due to our lack of awareness regarding the diagnosis of VCP, we decided to do an exploratory laparotomy.

At laparotomy, we observed a moderate amount of yellowish turbid fluid among the bowel loops, in the pelvis, and in the subhepatic area; diffuse fibrinous patches that cover the peritoneum and visceral organs; extensive adhesive exudate among bowel loops; and severely inflamed and thickened great omentum. We excluded the presence of any intra-abdominal injury by meticulous systematic exploration, which included the abdominal esophagus, stomach, duodenum, pancreas, small and large bowel, gallbladder, uterus, ovaries, ureters, and urinary bladder. Both ureters and bladder were found intact on intravenous urography and ascending urethrocystogram. We did plenty of peritoneal lavage with omental biopsy, and because the appendix was clinically questioned, we added an appendectomy. Postoperatively, the patient shifted to the intensive care unit (ICU) for close monitoring and respiratory support. The postoperative course was lengthy and complicated. Although the patient was on broad antibiotics (tigecycline, fluconazole, levofloxacin), high-flow oxygen, and chest physiotherapy, she persistently had tachycardia, tachypnea, dyspnea, fever, leukocytosis, abdominal pain, distention, tenderness, and increase oxygen demand. The workup of the third postoperative day revealed negative bacterial culture (blood, urine, and sputum). CT abdomen showed a new fluid collection anterior to the right lobe of the liver and CT chest revealed bilateral lung infiltrates and consolidation, which suggest an acute respiratory distress syndrome (ARDS). There was no DVT on venous doppler of both lower extremities, and the histopathology report diagnosed the case as VCP. Intravenous steroids (methylprednisolone 30 mg every 12 hours for seven days) were added to the current medications. After commencing the steroids, our patient had gradual and significant improvement over the subsequent days, and she was discharged home on the 10th postoperative day. Surprisingly, after two days, she presented to the emergency room complaining of blood-stained secretions from the laparotomy wound. On local wound exploration, we observed full wound dehiscence. Hence, the patient was transferred to the operating room; formal abdominal closure under general anesthesia was done. The patient was discharged home after five days of an eventless hospital course. We have been following up with our patient regularly, for six months and, fortunately, she is free of significant complications, apart from intermittent crampy abdominal pain.

**Figure 4:**
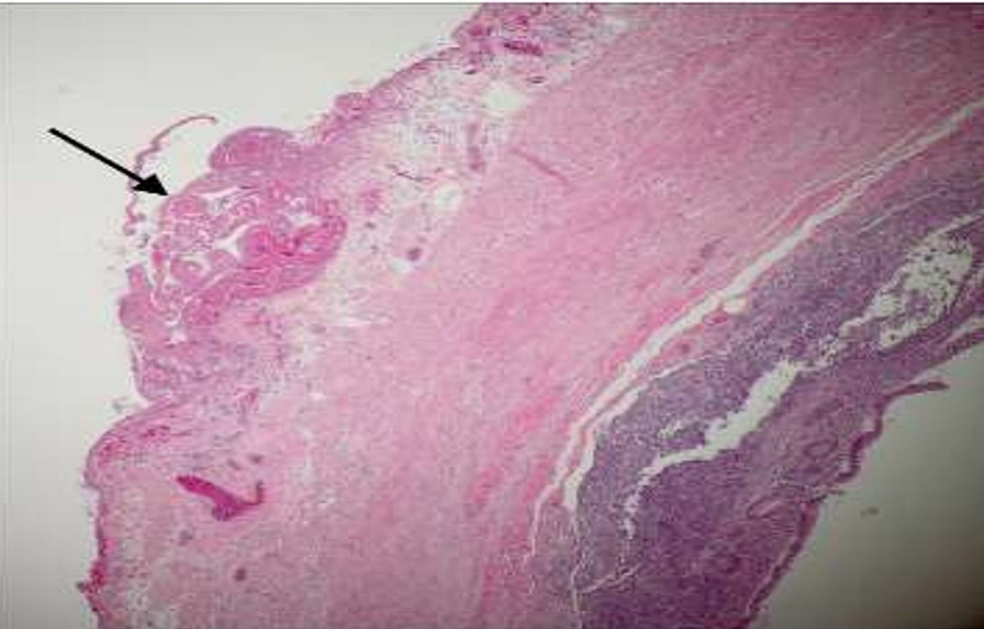
Appendix with keratin deposits in the surface of the serosa (arrow) Hematoxylin-eosin, X10

**Figure 5:**
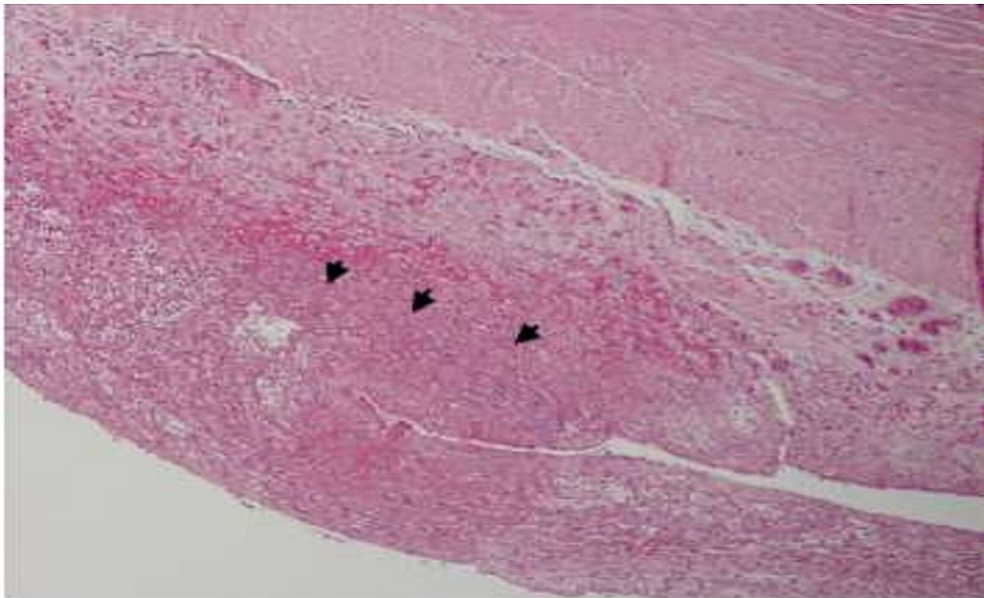
In the appendix serosa, there is an inflammatory infiltrate centered around the focal area of fetal squamous and keratin deposits (arrows heads) highly suggestive of vernix caseosa peritonitis Hematoxylin-eosin, X40

**Figure 6:**
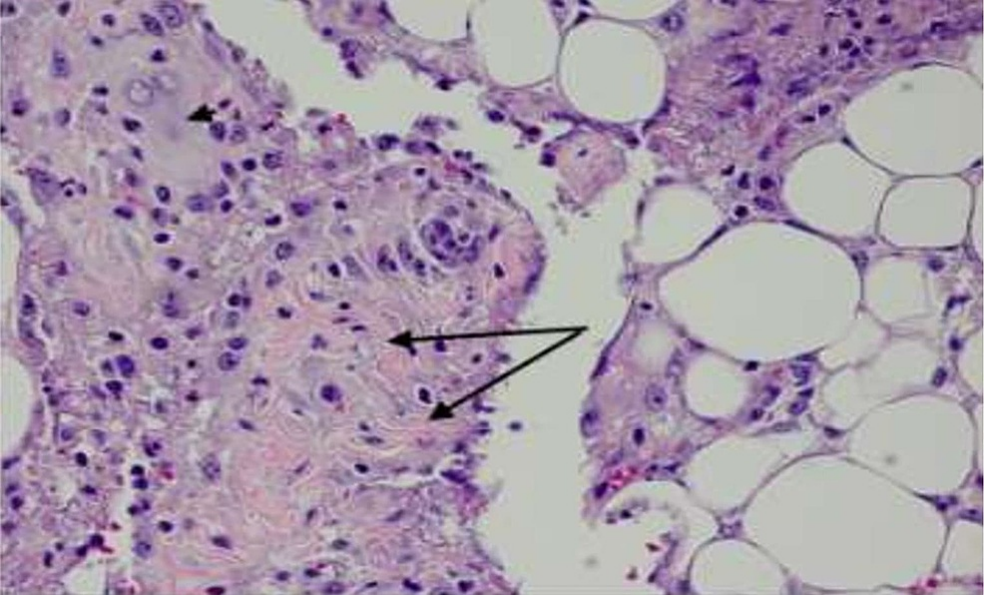
Omentum showing a dense inflammatory reaction, surrounding keratin deposits (long arrows) with the presence of giant cells (arrowhead) Hematoxylin-eosin, X40

The pathologic analysis of the removed appendix showed an appendicular wall lined with non-atypic, cylindrical, and not ulcerated epithelium; these findings mean normal epithelium. The mucosa and underlying muscularis propria are within the normal histopathological features. However, in the serosa, there is a mild peri-appendicular inflammatory infiltrate centered around a focal area of fetal squamous and keratin deposits. The histopathologic diagnosis is peri-appendicular peritonitis with a focal area of fetal squamous deposits. It is highly suggestive of vernix caseosa peritonitis (Figure 4). The histopathologic examination of the omental biopsy revealed an omentum with dense inflammatory infiltrate. It is a mixed infiltrate composed of neutrophils, histiocytes, and a few giant cells centered on aggregates of anucleate fetal squamous cells. The diagnosis is omentum with important polymorphous, inflammatory infiltrate associated with fetal squamous deposits in the surface. These features are highly suggestive of vernix caseosa peritonitis (Figures 5-6).

## Discussion

VCP was first described in 1976. It is a rare peripartum condition and is thought to be secondary to the dribbling of fetal amniotic fluid into the maternal abdominal cavity. Typically, the amniotic fluid reaches the mother’s abdomen during CS or from uterine perforation [2-4]; alternatively, it reaches by backflow from the uterus during vaginal delivery or prenatally [1,7]. The presence of the amniotic fluid into the maternal cavity initiates an inflammatory reaction and subsequent peritonitis [5]. The exact underlying pathophysiology is still poorly understood, however, one of the theories is the hypersensitivity reaction due to either antenatal or from previous pregnancy sensitization [8].

Clinically, VCP manifests hours to weeks after CS or vaginal delivery. Frequently, patients have signs and symptoms simulating acute abdomen: diffuse abdominal pain, distention; tenderness and rebound tenderness; fever (38.8°C); tachycardia (100-110/min) [6,8-9]. Shortness of breath and ascites can be part of the clinical picture [1-2,4]. The laboratory workup may reveal only an elevation of white cell count. Typically, all bacterial cultures are negative [3].

Radiologically, X-ray, ultrasound, CT, and MRI of the abdomen are normal or inconclusive [3]. The commonest findings on CT are ascites, multiple peritoneal cystic nodules mainly around the liver, free fluids in all abdominal regions, abscess formation or mass in the right lower quadrant, and inflammatory mass in the CS wound [2,6].

Numerous intraoperative findings were reported: a variant amount of bloody ascites, turbid fluids, yellowish plaques on the liver surface, extensive adhesions, fibrinous omental plaques, granulomatous inflammation, mass lesions resembling bowel perforation, and widespread white adhesive exudate covering the surface of the abdominal organs and parietal peritoneum. In most cases, no uterus or bowel perforation nor obvious intra-abdominal emergencies can be detected [1-4,6,9-10].

In the literature, the treatment of VCP falls into three categories: first, ultrasound-guided aspiration of the intraabdominal fluids and conservative treatment. Second, laparoscopy, peritoneal or omental biopsy, abdominal lavage, and intravenous antibiotics with steroids [2]. Third, laparoscopy or laparotomy, removal of questionable abdominal organs (appendix, ovary, bowel, colon, and gallbladder), which were found normal on histopathology study [3,5-6,10].

The histopathology examination of biopsies from the omentum, ascitic fluids, and peritoneum have numerous findings. These findings are basically influenced by the time between the amniotic fluid spillage into the peritoneal cavity and the microscopic examination [1,3]. In the early phase, only features of acute inflammation can be seen, both acute and foreign body inflammation in the middle phase, and mainly granulomatous foreign body inflammation in the late phase [1,3]. Thus, the histopathology report may reveal the presence of neutrophils, mixed cellular infiltrate, giant cells, fibrin, fetal hair, and squamous cells [1,6,9].

Both our study and literature share a number of key points: acute peritonitis following CS, indeterminate findings of diagnostic imaging, no clinical improvement on antibiotic therapy, and lack of awareness regarding the diagnosis among health providers. All these points made us suspect an intra-abdominal emergency and subsequent laparotomy with removal of the questioned appendicitis. On the other hand, our case is unique because, to our best of knowledge, this is the first reported and published case of VCP in Saudi Arabia, as the majority of reported cases are from the USA and a few are from the UK [6]. This case is important because VCP is less published in the literature. There was significant improvement after starting intravenous steroid therapy. The case has provided a deeper insight into VCP, and it adds to a growing body of literature on this rare entity. However, our case has two limitations: first, we could not utilize the laparoscopic approach because the on-call consultant has no experience with laparoscopy. Second, we forgot to take documentary photos of intraoperative findings.

Therefore, the clinical diagnosis of VCP should be kept in mind in patients who present with an acute abdomen following CS, particularly when the findings on CT abdomen are inconclusive and there is no improvement on antibiotics alone. Nevertheless, CT abdomen is the most helpful imaging modality. Peritoneal, omental, and ascitic fluid biopsies are diagnostic [2-3]. Once the diagnosis is confirmed, peritoneal lavage with intravenous antibiotics and steroids is the key foundation for successful management [2-3,6]. Considering the possibility of delayed complications, such as bowel obstruction and extensive adhesions that may occur in VCP cases, cautious monitoring is essential [9].

These data may help us create the following cautious strategy when dealing with post-CS acute abdomen: First, it is critical to maintain the diagnosis of VCP. Second, if the laboratory and imaging results are inconclusive, an image-guided aspiration of the intraabdominal fluid for histopathological evaluation could be undertaken. If the diagnosis of VCP is confirmed by a histopathological study, conservative treatment (intravenous antibiotics and steroids) will suffice. Third, if the intraabdominal fluid is not accessible, diagnostic laparoscopy is recommended. Intraoperatively, the presence of characteristic findings without any injury suggests the diagnosis of VCP; thus, the therapeutic options include fluid, omental, or peritoneal biopsy with peritoneal lavage. This approach might be useful in reducing the frequency of unnecessary laparotomies and normal organ removal.

## Conclusions

Despite its rarity, VCP is a serious complication of CS. Frequently, it is misdiagnosed and undervalued since it mimics the other types of acute peritonitis. Therefore, it is crucial to increase awareness of this rare diagnosis among healthcare providers and include it in the differential diagnosis of post-CS acute peritonitis especially when the imaging studies are inconclusive. Having this awareness may help doctors avoid unnecessary laparotomies and the removal of normal abdominal organs. Further research is needed to determine the exact underlying relationship between peritonitis and the spillage of amniotic fluid into the mother's abdomen during CS and why it does not always occur.
